# Pharmacodynamics of Flucloxacillin in a Neutropenic Murine Thigh Infection Model: A Piece of the Puzzle towards Evidence-Based Dosing

**DOI:** 10.3390/antibiotics11081049

**Published:** 2022-08-03

**Authors:** Eveline E. Roelofsen, Brenda C. M. de Winter, Heleen van der Spek, Susan Snijders, Birgit C. P. Koch, Sanne van den Berg, Anouk E. Muller

**Affiliations:** 1 Department of Hospital Pharmacy, Haaglanden Medisch Centrum, 2512 VA The Hague, The Netherlands; e.roelofsen@haaglandenmc.nl; 2 Rotterdam Clinical Pharmacometrics Group, 3015 GD Rotterdam, The Netherlands; b.dewinter@erasmusmc.nl (B.C.M.d.W.); b.koch@erasmusmc.nl (B.C.P.K.); 3 Department of Hospital Pharmacy, Erasmus MC University Medical Center, 3015 GD Rotterdam, The Netherlands; 4 CATOR, Center for Antimicrobial Optimized Treatment Rotterdam, 3015 GD Rotterdam, The Netherlands; s.vandenberg@erasmusmc.nl; 5 Department of Medical Microbiology and Infectious Diseases, Erasmus MC University Medical Center, 3015 GD Rotterdam, The Netherlands; h.vanderspek@erasmusmc.nl (H.v.d.S.); s.snijders@erasmusmc.nl (S.S.); 6 Department of Medical Microbiology, Haaglanden Medisch Centrum, 2512 VA The Hague, The Netherlands

**Keywords:** PK/PD index, MIC-distribution, beta-lactam, PD-target

## Abstract

For decades, flucloxacillin has been used to treat methicillin-susceptible *Staphylococcus aureus* (MSSA). Little is still known about its pharmacodynamics (PD). The present study aimed to determine the pharmacokinetic (PK)/PD index and the PD-index value minimally required for efficacy. MICs of 305 MSSA isolates were measured to determine the wild-type distribution. The PD of 8 *S. aureus*, 1 *S. pyogenes*, and 1 *S. agalactiae* isolates were evaluated in a neutropenic murine thigh infection model. Two *S. aureus* isolates were used in a dose-fractionation study and a dose–response analysis was performed additionally in the in vivo model. Data were analyzed with a population PK and sigmoid maximum effect model. The end of the wild-type distribution was 1 mg/L. The percentage of time the unbound concentration was above MIC (%*f*T > MIC) was best correlated with efficacy. For *S. aureus*, median %*f*T > 0.25 × MIC required for 1-log reduction was 15%. The value for *S. pyogenes* was 10%*f*T > MIC and for *S. agalactiae* 22%*f*T > 0.25xMIC for a 1-log reduction. The effect of flucloxacillin reached a 2-log reduction of *S. aureus* at 20%*f*T > 0.25xMIC and also for *S. pyogenes* and *S. agalactiae*, a reduction was reached. These data may serve to optimize dosing regimens currently used in humans.

## 1. Introduction

Flucloxacillin is a narrow-spectrum beta-lactam antibiotic of the penicillin class. It has been in clinical use for many decades, primary in the treatment of methicillin-susceptible *Staphylococcus aureus* (MSSA) infections in all grades of severity. The indications include skin and soft tissue infections, bone and joint infections, bacteremia, and endocarditis. Especially in skin infections, empirical treatment with flucloxacillin might also target streptococci, such as *Streptococcus pyogenes*, as the clinical distinction between staphylococcal and streptococcal infection can be difficult. Furthermore, co-infections of *S. aureus* and *streptococci* can also occur.

The antimicrobial effect of all beta-lactam antibiotics is time-dependent and therefore best correlated with the pharmacokinetic (PK)/pharmacodynamic (PD) index %*f*T > MIC (percentage of time of the dosing interval that the unbound concentration is above the minimum inhibitory concentration). For the treatment of *Enterobacterales*, it has been shown in both animal studies and clinical studies that the minimum value of %*f*T > MIC for the beta-lactam antibiotic ceftazidime is 40–45% [1,2,3]. However, in the treatment of MSSA infections with flucloxacillin, the PK/PD index target values are still unknown.

In addition, very limited data are available on the susceptibility to flucloxacillin. According to the breakpoint table of the European committee of antimicrobial susceptibility testing (EUCAST) [4], isolates that are susceptible to cefoxitin are also susceptible to flucloxacillin. Therefore, separate breakpoints for flucloxacillin are not available. As a result, there are no data available on the EUCAST website on the wild-type distribution [5]. The epidemiological cut-off (ECOFF) value is currently unknown.

In clinical practice, different dosing regimens are used without proper knowledge on the PK and PD. In some hospitals, therapeutic drug monitoring is also used to optimize flucloxacillin treatment [6]. In order to establish the optimal regimen, knowledge of the PK/PD index target value and the MIC wild type distribution is crucial. 

Since these crucial data for flucloxacillin were still lacking, we determined the flucloxacillin MIC distribution in a collection of MSSA isolates from clinical samples. Furthermore, we determined flucloxacillin PK in infected mice, and performed dose–fractionation as well as dose–response studies in the murine thigh infection model. PK/PD target values were determined for eight MSSA isolates and for a *Streptococcus pyogenes* and a *Streptococcus agalactiae* isolate. The aim of the study was to determine the PK/PD target value for flucloxacillin in MSSA. To put the results into a clinical context, available population models from the literature were used to calculate the probability of target attainment for several dosing regimen. This information might help to optimize flucloxacillin dosing.

## 2. Results

### 2.1. Pharmacokinetics

The pharmacokinetics of the total concentrations of flucloxacillin in mice were best described by a one-compartment model with first order absorption and Michaelis Menten elimination with an combined residual error. As compared to the final model, a model with zero order elimination resulted in an increase of 182 point in OFV and a model with first order elimination with 71 points in OFV. Inter-individual variation (IIV) was found on the Michaelis Menten constant (KM). The bootstrap had 485 out of 1000 successful runs. All model estimates were within the 95% percentiles of the simulations of the bootstrap. The final parameter estimates of the final model and the results of the bootstrap are shown in Table 1. 

### 2.2. Plasma Protein Binding

The relationship between the total concentration in plasma and the free fraction was described by a trend-line in Excel for the total concentration range of 20.1–303.9 mg/L. Free concentrations measured in samples with total concentrations <20.1 mg/L were below the limit of quantification (LOQ) and discarded. The total concentration of 20.1 mg/L corresponds to a free concentration of 0.75 mg/L. For the calculations of PK/PD exposures the following formula was used: Y = 0.0005x + 0.0409 (R^2^ = 0.8173), in which Y is the free fraction and x the total concentration.

### 2.3. Determination of the PK/PD Index and the Magnitude of the Index Correlated with Efficacy

**Figure 1:**
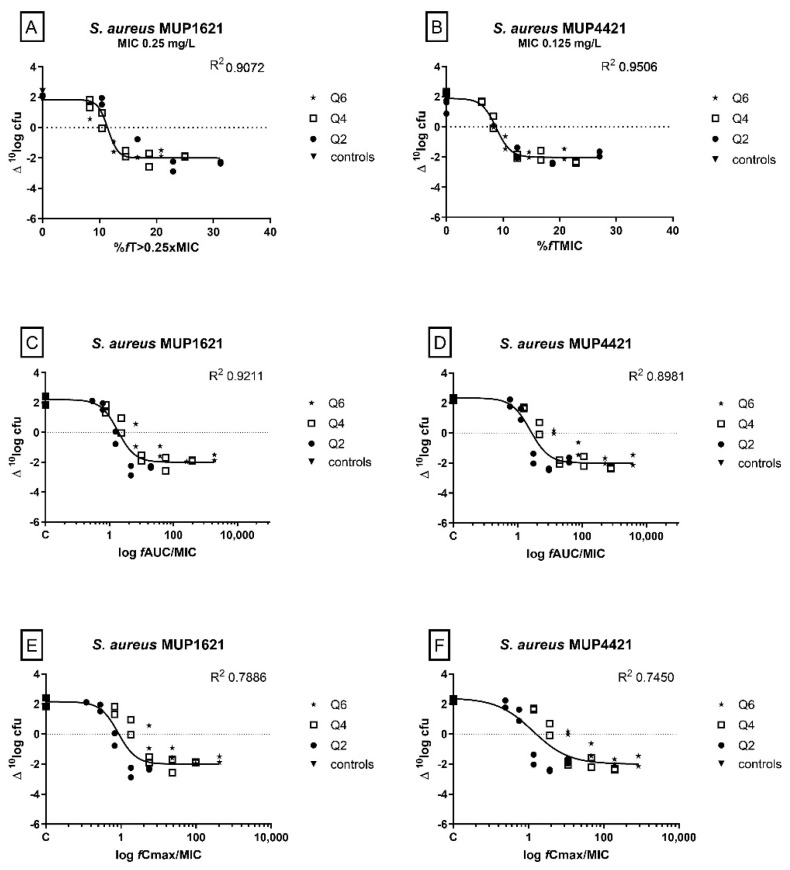
Relationships of flucloxacillin percentage of time the unbound concentration was above 0.25 times the MIC (%*f*T > 0.25xMIC) (**A**), %*f*T > MIC (**B**), 24 h *f*AUC/MIC (**C**,**D**), and *f*C_max_/MIC (**E**,**F**) for *S. aureus* MUP1621 and MUP4421 in the neutropenic thigh infection model, with change in CFU per thigh from start of treatment and after 24 h of treatments. Flucloxacillin was dosed every 2 (Q2), 4 (Q4), or 6 (Q6) h. Each symbol represents a therapy response in one mouse thigh. The line is the best-fit line based on the sigmoid maximum effect (E_max_) model.

In the dose–fractionation study of two *S. aureus* isolate, the %*f*T > MIC was best correlated with efficacy (%*f*T > 0.25xMIC; R^2^ 0.9072 for MUP1621 and %*f*T > MIC; 0.9506 for MUP4421). For the area under the unbound concentration–time curve divided by the MIC (*f*AUC/MIC), there was also a reasonable correlation with efficacy (R^2^ 0.9211 for MUP1621 and 0.8981 for MUP4421). While for MUP1621 the R^2^ was higher for the *f*AUC/MIC fit, visually the %*f*T > 0.25xMIC was better. The correlation with the maximum unbound concentration divided by the MIC (*f*C_max_/MIC) was clearly suboptimal (Figure 1).

**Figure 2:**
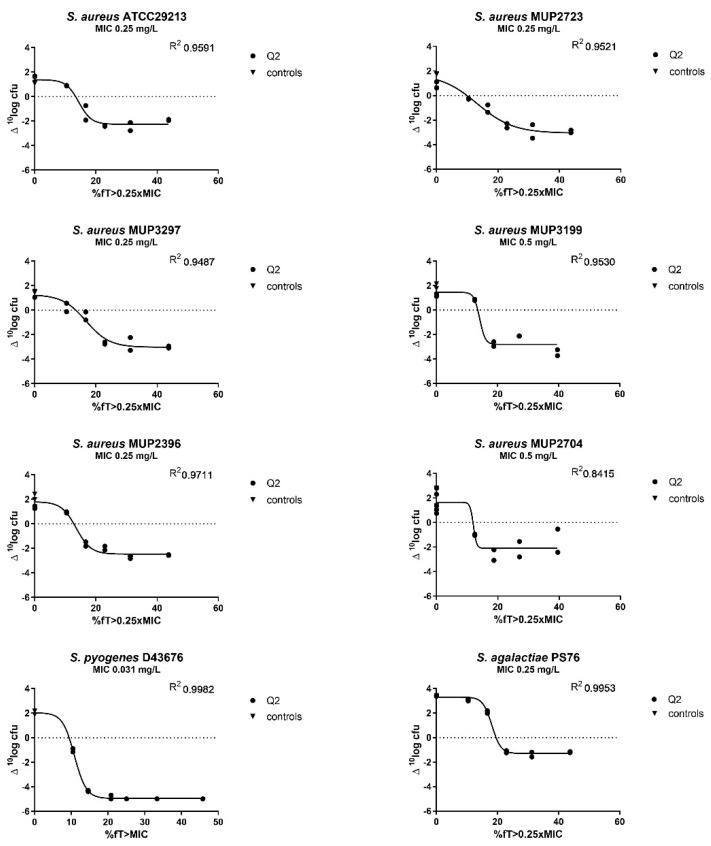
Dose–response of flucloxacillin against six *S. aureus*, one *S. pyogenes* and one *S. agalactiae* isolates in the neutropenic murine thigh infection model. Each symbol represents a therapy response in one mouse thigh. The *x* axis is the flucloxacillin exposure expressed as the percentage of time the unbound concentration was above 0.25 times the MIC (1 time the MIC for *S. pyogenes*). The *y* axis is the change in log_10_ of bacterial load from the start of treatment. The line is the best fit based on the sigmoid E_max_ model.

Dose–response studies were performed in a murine neutropenic thigh infection model with eight *S. aureus*, one *S. pyogenes* and one *S. agalactiae* isolates to estimate the %*f*T > MIC to result in a static, 1-log kill, and 2-log kill effect. The dose–response relationships were described by the sigmoid maximum effect (E_max_) model, and static 1-log and static 2-log reduction effects were calculated. The exposure–response curves of the individual isolates are shown in Figure 2. The %*f*T > MIC correlating with a static, 1-log and 2-log, kill for the individual isolates is presented in Table 2. For all *S. aureus* isolates, a 2-log kill effect was achieved at an average value of 20.0%*f*T > 0.25xMIC. For the *S. pyogenes* isolate, a 2-log kill effect was also achieved, while for the *S. agalactiae* isolate, only a 1-log kill was reached.

### 2.4. MIC Distribution

**Figure 3:**
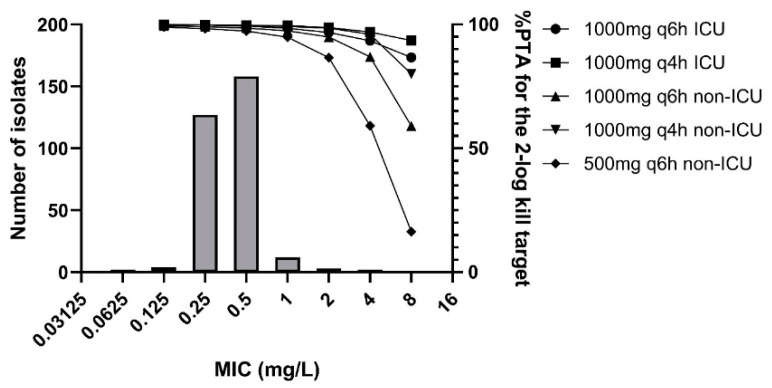
Distribution of MSSA isolates and the probability of target attainment (%PTA) for the 2-log kill target for several dosing regimens for intensive care unit (ICU) [7] as well as non-ICU hospitalized patients [8].

For a collection of 305 MSSA isolates, the flucloxacillin MIC distribution was determined and the results are shown in Figure 3. The 97.5% wild-type cut-off value for this selection of 305 isolates was 1 mg/L.

### 2.5. Monte Carlo Simulations

The probability of target attainment (PTA) for the range of MICs in different dosing regimens is shown in Figure 3. Taking into account the 2-log kill target and a MIC of 1 mg/L, the PTA in the non-ICU population reached values of 98.9% for 1 g q4h and 96.6% for 1 g q6h, which was slightly lower than the PTA of 99.8% and 99.7%, respectively, reached in the intensive care unit (ICU) population. In the non-ICU population, the PTA for a regimen of 500 mg was 91.6%.

## 3. Discussion

Flucloxacillin is a small-spectrum beta-lactam antibiotic, mainly used in the treatment of methicillin-susceptible *S. aureus* infections. For all beta-lactam antibiotics, the %*f*T > MIC is best correlated with efficacy, which was confirmed in the present study. The flucloxacillin protein binding in mice was non-linear, and the pharmacokinetics were best described in a population pharmacokinetic analysis by a one-compartment model with first-order absorption and Michaelis Menten elimination with an combined residual error and IIV on KM. The inclusion of Michaelis Menten kinetics in the model might be explained by the same mechanism as is seen in humans. In humans, it is known that it is predominantly excreted renally by glomerular filtration and tubular secretion. Tubular secretion is an active saturable process and might therefore explain the need for Michaelis Menten kinetics. 

The results of the PK model were used to calculate the PK/PD indices for the dose-fractionation and the dose–response curves for multiple *S. aureus* isolates, as well as for single isolates of *S. pyogenes* and *S. agalactiae*. A 2-log kill effect was reached for all *S. aureus* isolates with an average value of 20.0%*f*T > 0.25xMIC. The *S. agalactiae* isolate did not reach a 2-log kill effect but reached 1-log kill with a value of 22.1%*f*T > 0.25xMIC. This value for 1-log-kill in the *S. agalactiae* isolate seems to be slightly higher as compared to the *S. aureus* isolates. The E_max_ model for the *S. pyogenes* isolate was best described by the %*f*T > MIC and low percentages of time of the dosing interval were needed to reach 2-log kill in this single isolate. 

The values found for the MICs are in line with the values reported previously in literature, however large distributions are lacking. A study in 37 MSSA isolates reported values between 0.06 and 0.25 mg/L [9], while in case reports, higher values for the flucloxacillin MIC were reported of up to 1 mg/L [10,11]. Because of the lack of data on MIC distribution for flucloxacillin, several studies use MIC values for oxacillin instead with an ECOFF of 2 mg/L [12,13,14,15]. As it appears that the flucloxacillin MICs are lower compared to the oxacillin values, the target-attainment as reported in these trials might be too low. Results or conclusions of these studies might need to be reconsidered.

The flucloxacillin PK/PD target as found for *S. aureus* in the present study seems relatively low. For many other beta-lactams, such as amoxicillin/clavulanic acid and cefuroxime, the PK/PD target for *S. aureus* is not known. However, for the fifth-generation cephalosporins, the magnitude of the %*f*T > MIC predictive of cephalosporin efficacy to treat *S. aureus* ranges from 15–40% and 25–40%, for ceftobiprole and ceftaroline, respectively, depending upon the defined therapeutic endpoint (stasis to 2-log-unit reduction) [16,17,18]. For two broad spectrum carbapenems, including efficacy against *methicillin-resistant Staphylococcus aureus* (MRSA), the magnitude found for stasis was only 5 ± 1.4%*f*T > MIC for razupenem and 27% for tomopenem [19,20]. The PK/PD targets for Gram-negative bacteria is generally much higher (40–70%*f*T > MIC) [21,22].

From a clinical perspective, we expect flucloxacillin to be active at low values for the PK/PD target. The standard dosing regimen to treat cellulitis in the Netherlands is a 500 mg oral dose of flucloxacillin, four times daily. Most patients respond well to this regimen. In the literature, it was shown that after an oral dose of 1000 mg, the mean maximum unbound concentration in patients who are fed is approximately 0.55 mg/L, and the %*f*T > MIC_0.25_ is 58% [23]. Given the fact that the doses used in these patients are only 500 mg, the observed effect is in line with the low PK/PD target value found in the current study.

This study focused on the effect of flucloxacillin on *S. aureus*, since this is the most important micro-organism treated with flucloxacillin. However, in some instances, the effect on other micro-organisms might be relevant as well. For example, in clinical practice, the distinction between cellulitis (mainly caused by *S. aureus*) and erysipelas (mainly caused by *Streptococcus pyogenes*) is difficult, and therefore, flucloxacillin is initiated in many circumstances. To that end, we included a single isolate of *Streptococcus pyogenes* and *Streptococcus agalactiae*. This study showed that flucloxacillin was highly active against those isolates. However, to determine the target-value for those micro-organisms, more isolates should be included in the analysis.

The PK/PD index target value as found for *S. aureus* can subsequently be used to optimise dosing regimens. To that end, it is important to analyze unbound concentrations. Unfortunately, protein-binding of flucloxacillin is complex, as the degree of protein-binding in patients is unpredictable [13,15] and also depends on the serum albumin level [8]. Most pharmacokinetic data described are currently based on total concentrations, and there is a large variability between patients [24,25,26,27,28,29,30]. Two population PK models using unbound concentrations were used to calculate the PTA using the target value found in this study and showed that especially in ICU patients the PTA is high for both the 1 g q4h and q6h regimen. Also, in the non-ICU population a PTA of >95% was reached for both regimens. The higher PTA in the ICU population might be explained by the lower protein binding as compared to the non-ICU population.

In conclusion, this the first study that present the PK/PD target values correlated with bacterial kill for flucloxacillin and *S. aureus*. The end of the MIC distribution of a collection of MSSA isolates was found to be 1 mg/L. Taking these findings into account, dosing regimen of 1 g every 4 or 6 h seems adequate based on simulations of previously published models in ICU and non-ICU patients [7,8]. These PD-target values are a crucial piece of the puzzle towards evidence-based dosing, but other issues such as defining an epidemiological cut-off value, clarify the protein-binding in several patient populations and knowledge on tissue penetration are also of importance.

## 4. Materials and Methods

### 4.1. Bacteria, Media, and Antibiotics for Animal Experiments

In vivo experiments were performed with eight *S. aureus* isolates, one *S. pyogenes*, and one *S. agalactiae* isolate. Except for the *S. aureus* ATCC reference strain, all isolates were clinical isolates. A freezer stock of approximately 1 × 10^9^ colony forming units (CFU)/mL in MHB was prepared for all isolates. At the day of infection, log-phase cultures were made from this freezer stock in fresh Mueller–Hinton Broth (MHB) and incubated for 1 or 2 h (depending on the isolate) at 37 °C, under shaking conditions. The log-cultures were diluted with MHB (thigh) or physiological saline solution (lung) to a final inoculum of approximately 10^8^ CFU/mL for infection. The number of CFU during the experiments were counted on Mueller–Hinton II agar (MHA) plates (Becton Dickinson, Olen, Belgium). Commercially available flucloxacillin (Floxapen Aurobindo Pharma B.V., Baarn, The Netherlands) was used. Dilutions of flucloxacillin were prepared in saline one hour prior to treatment and stored at 4 °C until further use.

### 4.2. Animals

Dose–response experiments were performed in the Erasmus Laboratory Animal Science Center (EDC) in Rotterdam, the Netherlands, following the EU Animal Directive 2010/63/EU 2010 [31] with license number AVD101002016702. Seven- to eight-week-old outbred female CD-1 mice (body weight 25 ± 5 g at the day of infection) were obtained from Charles River (Germany). Mice were housed under standard conditions with food and water ad libitum. The mice were rendered neutropenic by intraperitoneal injection of two doses of cyclophosphamide at 4 days (150 mg/kg of body weight) and 1 day (100 mg/kg of body weight) before the onset of the experiment.

### 4.3. Thigh and Lung Infection Model in Neutropenic Mice

Infection in the thigh infection model was induced by intramuscular injection of 0.05 mL of approximately 10^8^ CFU/mL in each thigh. Pneumonia was induced in isoflurane-anesthetized mice by applying 0.05 mL of the inoculum intranasally. The inoculum was checked by plating 10-fold serial dilutions on MHA plates. In all experiments, the lower limit of detection was 10 CFU/tissue.

Antimicrobial treatment was started 2 h after the mice were infected in the thighs or lungs. Doses of flucloxacillin were injected in 0.1 mL saline subcutaneously. Single doses of 1–128 mg/kg were administered to determine the pharmacokinetics, and all doses were studied in duplicate. For the pharmacodynamics dosing regimen of 2–64 mg/kg administered every 2 h were used. Additionally, for two *S. aureus* isolates, a second (8–128 mg/kg every 4 h) and third regimen (32–256 mg/kg every 6 h) were studied.

To determine the pharmacokinetics, blood samples were taken under isoflurane anaesthesia through orbital sinus bleeding from infected mice. Blood samples were taken before (t = 0) and after (t = 5, 10, 20, 30, 40 min, 1, 1.5, 2, 4, and 6 h) single administration of flucloxacillin. Blood samples were collected in K3E EDTA tubes (Sarstedt, Nümbracht, Germany) and centrifuged immediately at 15.871 rcf for 5 min at 4 °C in a precooled centrifuge to separate plasma. Plasma was stored at −80 °C until analysis.

Pharmacodynamics was studied in the thigh infection model. At start of treatment, two mice were humanely euthanized to determine the bacterial load in the thighs. After 24 h of treatment the remaining mice were humanely killed for determination of CFU counts. The thighs were excised and homogenized in 2 mL of phosphate buffered saline (PBS) by using a T-25 Ultra-Turrax instrument. Bacterial burden in the thigh infection models were quantified by culturing 10-fold serial dilutions of the homogenized tissue on MHA plates.

### 4.4. Measurement of Flucloxacillin Concentrations in Plasma

Total and unbound flucloxacillin plasma concentrations were measured using a multi-analyte UPLC-MS/MS assay [32], validated in accordance with the FDA guidance on bioanalytical method validation [33]. The method has been developed and validated in the pharmacy department of the hospital in which most of the authors are employed. Samples above the linearity of the calibration curves for both total and unbound concentrations (1.0–123.0 mg/L R^2^ > 0.99) were diluted according to standard dilution protocol. To determine unbound concentrations plasma was centrifuged for 12 min at 14,680 RPM before analysis. Free concentrations were obtained after temperature-controlled ultrafiltration of 200 µL of plasma using Nanosep 30 K Omega Centrifugal Devices (VWR International B.V., Amsterdam, The Netherlands). Then, 50 µL of the filtrate was used for the sample preparation and measured as previously described [32].

### 4.5. Flucloxacillin Pharmacokinetics in Mice

To describe the pharmacokinetics of the total concentrations, a population model was developed using nonlinear mixed-effects modeling (NONMEM, version 7.4.2, ICON Development Solutions, Ellicott City, MD, USA). The graphical user interface Pirana (version 2.9.7; Certara, Phoenix, AZ, USA) was used in combination with R Studio (version 1.1.463; RStudio, Boston, MA, USA), R (version 3.5.2; R-core team, Vienna, Austria), Xpose (version 4.6.1; Department of Pharmaceutical Biosciences, Uppsala, Sweden), and PsN (version 4.8.1; Department of Pharmaceutical Biosciences, Uppsala, Sweden) for the selection and evaluation of the model, objective function, goodness of fit plots (GOF), and bootstrap (*n* = 1000) were used. For the objective function a difference of 3.84 points (*p* 0.05) was considered significant. The individual and population predictions were plotted versus the observed concentrations and evaluated for the distribution around the line of identity. The model estimates must be within the 95% confidence interval of the simulations of the bootstrap.

In the modeling procedure several structural models, statistical models, error models and approaches for handling LOQ were tested. Samples below the LOQ were calculated as 0.5 mg/L with an additional error up to the first time point in which both samples were below LOQ. Successive measurements were discarded but confirmed after model development by simulating the concentration. The final model was presented assuming a 100% bioavailability and for a 1 kg mouse.

### 4.6. Pharmacodynamic and Statistical Analysis

To determine the relationship and evaluate a potential concentration dependency between the total concentration and the free fraction (fu), the values were plotted and described by a trend-line in Excel (Microsoft Corporation, 2019). The population PK model was used to calculate the %*f*T > MIC, *f*AUC/MIC, and *f*C_max_/MIC over a period of 24 h in NONMEM for the different dosing regimen and a range of MIC values.

Dose–fractionation studies were performed for 2 *S. aureus* isolates (MUP1621 and MUP4421). Regimens every 2 h, 4 h, and 6 h were used and the corresponding values for the *f*AUC/MIC, *f*C_max_, and %*f*T > MIC were calculated. The different values for the three PK/PD indices were subsequently fit to the CFU data by using a sigmoid maximum-effect (E_max_) model with a variable slope (Graphpad Prism, version 7.0, GraphPad, Inc., San Diego, CA, USA). Goodness-of-fit and the R^2^ values were compared.

Pharmacodynamic-pharmacokinetic dose-response curves were fit to the CFU data by using a E_max_ model with a variable slope (Graphpad Prism, version 7.0, GraphPad, Inc., San Diego, CA, USA) to determine the effect of flucloxacillin on change in colony counts. To choose the best fit the goodness-of-fit and the R^2^ values were compared. The static PK/PD index was calculated by substitution of the bacterial tissue load at start of the treatment by the use of an E_max_ model. Subsequently, PK/PD indices for stasis, 1 log10-kill, and 2 log10-kill were determined.

To place the PK/PD target value found for *S. aureus* in clinical perspective, simulations were performed with two previously published PK models describing the PK of unbound concentrations: ICU patients [7] and non-ICU hospitalized patients [8]. Briefly, both the models of Wilkes and Wallenburg were based on two-compartments and include an overall proportional residual error as well as a separate proportional residual error on the unbound concentrations [7,8]. They also both estimate a dissociation constant (KD) and a maximum binding capacity (Bmax) for the protein binding. The Wilkes model described the flucloxacillin clearance including the CKD-EPI creatinine and cystatin C equation (CKDCRCYS) as follows: Θ_clearance_ + (CKDCRCYS − 90) * Θ_renal_. While in the Wallenburg model the 24 h urinary CL_cr_ was included as follows: ΘCL_non-renal_ + urinay 24 h CL_cr_ * ΘCL_renal_. Both models have interindividual variability (IIV) on clearance and the central volume of distribution, but the Wallenburg model had an additional IIV on Bmax. Since in the Wallenburg study the samples were taken on two occasions within 48 h, an inter-occasion variability was included on clearance. In both models the bound concentrations was described as (concentration unbound * Bmax)/(KD + unbound concentration). In the Wilkes model Bmax was influenced by serum albumin: 1 + (Θ_impact of albumin on Bmax_ * (serum albumin − 25.6)), while in the Wallenburg model the influence of albumin on Bmax was described differently (Bmax = Θ_Bmax_ + serum albumin * Θ_Bmax slope_). The PTA for the 2-log kill targets in both populations were determined. Monte Carlo simulations (*n* = 5000) were performed on steady state concentrations with their final models using NONMEM for several regimen (doses infused over 15 min). Covariates were set om median values. For ICU patients, fat-free mass was set at 58.2 kg, albumin concentration at 18 g/L and 24 h creatinine clearance at 65 mL/min. For non-ICU patients, albumin was set at 25.6 g/L and CKD-EPI based on creatinine and cystatin C at 90 mL/min.

### 4.7. Determination of Minimum Inhibitory Concentrations (MIC) and Wild-Type Distribution

All MICs were determined by standard microdilution procedures with geometric two-fold serial dilutions in Mueller–Hinton II broth (MHB; Becton Dickinson, Olen, Belgium) according to the guidelines of EUCAST. A collection of 305 MSSA isolates obtained from clinical samples from different hospitals around the Netherlands was used to determine the distribution of MIC values and the end of the wild-type distribution. For these isolates, the MIC was determined a single time, while for the isolates used in studies in mice, the MICs were determined in triplicate. The median value of the replicate MICs for the in vivo experiments was reported and subsequently utilized in PK/PD analyses. The end of the wild-type distribution of the selection of 305 MSSA isolates was determined by using the ECOFFinder (ECOFFinder_XL_2010_v2.1_web-version, available on the EUCAST-website).

## Figures and Tables

**Table 1:** Final estimates and bootstrap.

	Model		Bootstrap	95% Percentiles	
Parameters	Estimate	Rel. SE (%)	Median	2.5%	97.5%
Ka (h^−1^)	9.78	28	9.59	6.09	19.48
V (L)	0.511	17	0.51	0.31	0.70
KM (mg/L)	7.51	18	7.36	3.99	10.67
Vmax (mg/h)	98.8	4	98.34	45.65	131.45
Variability on KM (%)	41.2	60	40.7	4.1	69.5
Additive error (mg/L)	0.0477	4	0.048	0.047	0.049
Proportional error (%)	42.5	16	40.0	22.4	56.8

Ka: absorption rate constant; Vmax: maximum velocity; KM: Michaelis Menten constant, V: volume of distribution, Rel.SE: relative standard error; CI: confidence interval.

**Table 2:** Values for the PK/PD index for the individual isolates and the average, median, and SD for the eight *S. aureus* isolates, one *S. agalactiae* and one *S. pyogenes* isolate.

Isolates	Stasis	1-log Kill	2-log Kill
%*f*T > 0.25xMIC			
*S. aureus* ATCC29213	13.38	15.71	19.73
*S. aureus* MUP4421	13.77	15.29	19.18
*S. aureus* MUP1621	11.42	12.41	30.60
*S. aureus* MUP2723	9.92	14.90	20.64
*S. aureus* MUP3297	13.44	17.20	22.21
*S. aureus* MUP3199	13.53	14.41	15.47
*S. aureus* MUP2396	12.79	15.10	18.58
*S. aureus* SA2704	11.91	12.50	13.95
*S. aureus*-average	12.52	14.69	20.05
*S. aureus*-median	13.09	15.00	19.46
*S. aureus*-SD	1.34	1.60	5.04
*S. agalactiae*	19.55	22.13	n.a.
%*f*T > MIC			
*S. pyogenes*	9.39	10.41	11.34

LogKill: reduction in number of CFU expressed in logarithm, MIC: minimum inhibitory concentration, SD: standard deviation, n.a.: not achieved.

## Data Availability

The data presented in this study are available on request from the corresponding author. The data are not publicly available due to local policies.
